# To Ascertain the Power of a Microscope

**Published:** 1869-03

**Authors:** 


To Ascertain the Power of a Microscope.—The *Scientific American* gives the following method for this purpose:

Place a small object of known length, say from 1-20th to 1-50th of an inch, on the stage of the microscope, and looking at this through the instrument with one eye, with the other look at a foot rule held at the level of the stage. With a little practice both may be seen at once, when by dividing the space apparently occupied by the object on the scale by the known length of the object, the magnifying power will be obtained.

If the power is very high, the best object to use is a glass micrometer, which may be purchased for a dollar or two, of any optician, with lines ruled on it to hundredths or thousandths of an inch.

